# Understanding mental health help-seeking and stigma among Hungarian adults: A network perspective

**DOI:** 10.1192/j.eurpsy.2024.1772

**Published:** 2024-09-19

**Authors:** Valerie S. Swisher, Dorottya Őri, Zoltán Rihmer, Róbert Wernigg

**Affiliations:** 1 The Pennsylvania State University, State College, University Park, PA, USA; 2 Institute of Behavioural Sciences, Semmelweis University, Budapest, Hungary; 3 Department of Psychiatry and Psychotherapy, Semmelweis University, Budapest, Hungary; 4 Nyírő Gyula National Institute for Psychiatry and Addictions, Budapest, Hungary; 5 National Directorate-General for Hospitals, Budapest, Hungary

**Keywords:** experiential avoidance, help-seeking behavior, Hungary, network analysis, public stigma, self-stigma

## Abstract

**Background:**

Hungarians exhibit more negative attitudes toward help-seeking for mental health problems compared to other European countries. However, research on help-seeking in Hungary is limited, and it is unclear how stigma relates to help-seeking when considering demographic and clinical characteristics. We used a network analytic approach to simulate a stigma model using hypothesized constructs in a sizable sample of Hungarian adults.

**Methods:**

Participants were 345 adults recruited from nine primary care offices across Hungary. Participants completed self-report measures assessing public stigma, self-stigma, experiential avoidance (EA), attitudes toward seeking professional psychological help, anxiety, depression, demographics, prior use of mental health services, and whether they have a family member or friend with a mental health condition.

**Results:**

EA and anxiety were the most central nodes in the network. The network also revealed associations between greater EA with greater public stigma, anxiety, depression, and having a family member or friend with a mental health condition. More positive attitudes toward seeking help were associated with lower self-stigma, public stigma, and having received psychological treatment in their lifetime. Being female was associated with lower income, higher education, and having received psychological treatment in their lifetime. Finally, having a family member or friend with a mental health condition was associated with having received psychological treatment in their lifetime and greater public stigma.

**Conclusions:**

The strength centrality and associations of EA with clinical covariates and public stigma implicate its importance in stigma models. Findings also suggest that while some aspects of existing stigma models are retained in countries like Hungary, other aspects may diverge.

## Introduction

Despite an increased demand for mental health services [1], up to 74% of people experiencing mental illness do not receive treatment in Europe and the USA [2, 3]. Stigma toward mental illness, or the process by which people with mental illness experience implicit or explicit social rejection, is one barrier that contributes to reduced help-seeking [4, 5]. However, while research on stigma has grown in the last few decades, it largely focuses on American and Western European populations, neglecting other countries that may exhibit different beliefs and values. One example is Eastern Europe, which exhibits some of the highest annual suicide rates worldwide [6, 7]. Hungary, in particular, exhibited the highest suicide rate in the world for the majority of the 1960s to 2000s [5, 7]. Despite this mental health crisis, research on mental health-related stigma is largely limited in Hungary.

Population survey research found that Hungarians exhibit more negative attitudes toward help-seeking for mental health problems and the highest levels of personal stigma relative to participants in Germany, Portugal, and Ireland [8, 9]. Additionally, research on post-communist countries suggests that people with mental illness may experience more discrimination in these locations [10]. This aligns with research on stigma toward mental illness among psychiatrists, in which Hungary was in the middle between Western European and Eastern European countries based on their stigma scores [11]. Importantly, despite improved mental health services and initiatives across Hungary in recent years (e.g., [12–15]), serial cross-sectional research suggests that there has been no meaningful change in attitudes toward people with mental illness in Hungary [16].

While research on stigma in Hungary is limited, research on stigma in other populations can inform conceptualizations of a stigma model among Hungarians. Individuals experience various types of stigma, including *self-stigma*, expressed as shame toward oneself, and *public stigma*, defined as perceived societal stereotypes toward mental illness, which both can hinder help-seeking [17–19]. The Internalized Stigma Model suggests that public stigma is internalized as self-stigma over time, predicting help-seeking behavior [17, 20, 21].

Demographic variables, including gender, age, and socioeconomic status, have been examined as predictors of stigma and mental health help-seeking, though the findings are mixed. Men tend to have more negative attitudes toward seeking help, underutilize services, and experience greater self and public stigma relative to females [22, 23], though other studies find that females report higher perceived stigma relative to males [24–26]. Regarding age, some studies find that older adults demonstrate more positive attitudes toward help-seeking and lower stigma relative to younger adults [22, 27], though findings remain mixed [28, 29]. Research also suggests that higher education is associated with decreased stigma [30], but higher income is associated with greater stigma [31], with some researchers suggesting that higher income individuals may be exposed to resource-rich environments that contribute to a greater likelihood of perceiving mental illness as controllable [31].

Experiencing mental health symptoms oneself and exposure to others with mental health conditions are other important predictors of stigma and help-seeking. One study found that contact with people with mental illness improved attitudes toward people with mental illness [32]. Similarly, greater contact and knowledge of mental illness predicted lower levels of personal stigma toward anxiety and depression [33]. However, familiarity with mental illness may not always attenuate stigma. Family members of those with mental illness may experience increased burden, which may lead to internalized stereotypes and increased self and public stigma [34]. Additionally, family members or close friends may experience courtesy stigma, or stigma received due to being associated with someone with mental illness, which may lead family members to have more negative attitudes toward mental illness, thereby increasing stigma. Lastly, personal experience with symptoms may worsen stigmatizing attitudes. Studies have found that those experiencing anxiety and depression were more likely to have greater perceived and self-stigma [33], and depression severity moderated the relationship between anticipated stigma and treatment seeking over time [35].

Finally, experiential avoidance (EA), or attempts to avoid experiencing unpleasant emotions, thoughts, or feelings, has been examined as a contributor to stigma and reduced help-seeking behavior. EA is a transdiagnostic feature of and highly correlated with emotional disorders (e.g., anxiety, depression) [36] and is associated with increased self-stigma [37]. EA may serve as both a maladaptive coping strategy against stigmas (e.g., avoiding unpleasant feelings associated with experiencing stigma) and as a barrier to help-seeking (e.g., avoiding unpleasant feelings that therapy may evoke). Applications of this conceptualization have found that self-stigma mediates the relationship between public stigma and intentions to seek help, and that this mediation model is moderated by EA [38]. These authors conclude that, as existing interventions to reduce public and self-stigma have mixed efficacy (e.g., [32, 39, 40]), targeting EA may be a novel approach to reducing stigma and increasing help-seeking [38]. Therefore, the present study used a network analytic approach to simulate a stigma model using hypothesized constructs in a sample of Hungarian patients visiting their primary care providers. Network analysis allows for the simultaneous examination of the partial correlations among all variables to delineate the importance, or strength, of each variable in the network without specifying outcome and predictor variables. Identifying the variable with the greatest strength may be helpful in identifying targets for future anti-stigma interventions. Thereby, network analysis is a data-driven, exploratory approach that allows us to visualize and understand the complex relationships between variables. We examined the association between demographic (age, gender, income, education), stigma (self-stigma, public stigma, attitudes toward seeking help), exposure to mental health (receiving psychological treatment in their lifetime, having a close family member or friend with a mental health condition), and clinical (anxiety, depression, EA) variables to examine the most central nodes (i.e., nodes with the most connections to all other nodes in the network) and relevant associations in a stigma network. In line with research indicating the relevance of EA to both stigma and clinical symptoms, we hypothesized that EA would be the most central node in the network.

## Method

### Participants

Participants were 345 adults (214 females, 62%), ages 18–85 (M = 46.37, SD = 14.62), recruited from nine primary care offices across four counties of Hungary (Budapest, Heves, Pest, and Borsod-Abaúj-Zemplén). Between November 2023 and February 2024, a member of the research team visited each office at least once and invited all visiting patients to partake in the survey, either by paper, pencil, or online (via Qualtrics). In addition, four of the nine participating primary care doctors emailed survey links to their patient listserv. Participants agreeing to participate completed measures assessing demographics, personal experiences with mental health problems, public stigma, self-stigma, EA, attitudes toward seeking help, anxiety, and depression. Of the 361 patients who consented, 6 were excluded for incompleteness (i.e., completed only the demographics or less) and 10 were excluded due to failed attention checks (e.g., answered anything other than “Very Much So” when asked “Please selected “Very Much So” for this question.”), resulting in a final sample of 345 participants. All study procedures were approved by the local National Scientific and Ethical Committee (TUKEB # BM/30518-1/2023).

One hundred and eleven (32.2%) participants reported having received treatment for their psychological or mental health problems in their lifetime and 52 (15.1%) reported receiving help in the last 12 months. Significantly more females (*n* = 83) endorsed having received treatment for their psychological or mental health problems in their lifetime relative to males (*n* = 28), *Χ*^2^ (1, *N* = 344) = 10.23, *p* = 0.001. Additionally, 37.7% (*n* = 130) endorsed having a close family member or friend with a mental health condition. Out of those who endorsed receiving treatment in their lifetime (*n* = 111), 58 (52.3%) endorsed receiving psychotherapy or counseling and 41 (36.9%) endorsed taking medication. Participants endorsed receiving anxiolytics (*n* = 21), anti-depressants (n = 15), anti-psychotics (n = 1), and other (n = 5; e.g., “Antihistamines”). See Table 1 for all sample characteristics.

**Table 1.** Sample demographics and use of psychological services

Descriptive	*n*	%*
Mean age (years, ± SD)	46.37 (14.62)	
Sex		
Male	130	37.7%
Female	214	62.0%
Unanswered	1	0.3%
Ethnicity		
Hungarian	336	97.4%
Roma	6	1.7%
Other (e.g., Germanic)	3	0.9%
Occupation		
Employed	262	75.9%
Unemployed	6	1.7%
Student	13	3.8%
Retired	51	14.8%
Other	12	3.5%
Unanswered	1	0.3%
Education		
Less than 8 overall	1	0.3%
8 general	16	4.6%
Vocational training without high school diploma	49	14.2%
Graduated	27	7.8%
Graduated and vocational education	102	29.6%
College or university bachelor’s degree	97	28.1%
University Master’s degree	42	12.2%
Postgraduate education	10	2.9%
Unanswered	1	0.3%
Income		
Less than 232 000 HUF	48	13.8%
232 000 HUF – 450 000 HUF	147	42.6%
450 000 HUF – 566 800 HUF	59	17.1%
More than 566 800 HUF	71	20.6%
Unanswered	20	5.8%
Town		
Capital (Budapest)	121	35.1%
County seat, town with county rights	26	7.5%
Other city	136	39.4%
Village	62	18.0%
County (Participant’s Residence)		
Borsod–Abaúj–Zemplén	51	14.8%
Budapest	96	27.8%
Borsod–Abaúj–Zemplén	51	14.8%
Budapest	96	27.8%
Heves	75	21.7%
Fejér	1	0.3%
Pest	119	34.5%
Szabolcs–Szatmár–Bereg	1	0.3%
Veszprém	1	0.3%
Unanswered	1	0.3%
Received help in last 12 months		
Yes	52	15.1%
No	291	84.3%
Prefer not to say	2	0.6%
Received help in lifetime		
Yes	111	32.2%
No	231	67.0%
Prefer not to say	3	0.9%
Family/friend with mental health condition		
Yes	130	37.7%
No	203	58.8%
Prefer not to say	10	2.9%

*Note*: Percentages are out of all 345 participants including those who left the question unanswered.

### Measures

#### Demographics and experience with mental health

Participants completed a questionnaire assessing age, gender, ethnicity, occupation, education, income, residence (i.e., town, county), and personal experiences with mental health. Personal experience with mental health was assessed using a yes (0) or no (1) response option to the questions, “In [your lifetime/the past 12 months], did you think you needed help for emotional or mental health problems such as feeling sad, blue, anxious, or nervous?” and “In [your lifetime/the past 12 months], have you received any treatment for emotional or mental health problems (e.g., therapy, counseling, medication)?” To assess experience with mental health via a close friend or family member, participants were asked, “Do you have a friend or family member who is experiencing mental illness?” Participants who endorsed receiving mental health treatment in their lifetime were asked to specify the treatment type (e.g., Psychotherapy, Medication, Other) and were allowed to select multiple responses (i.e., both medication and psychotherapy). Participants selecting “Medication” were given an open-ended response option to specify the type of medication received. Open-ended medication responses were coded into four categories: anti-anxiety (e.g., Benzodiazepines); selective serotonin reuptake inhibitors (SSRIs; e.g., Escitalopram); antipsychotic (e.g., Haloperidol); and other.

#### Attitudes toward seeking professional psychological help-short form (ATSPPH-S)

The ATSPPH-S [41] is a 10-item questionnaire measuring attitudes toward help-seeking for psychological problems. Items are rated from 0 (Disagree) to 3 (Agree), with higher scores indicating greater openness and more positive attitudes toward help-seeking. For example, participants were asked, “If I thought I was having a mental breakdown, my first thought would be to get professional attention.” It was translated into Hungarian by Coppens and colleagues (2013), and permission was obtained to use the questionnaire in the present study. Prior studies suggest that the ATSPPH-SF exhibits good test–retest reliability and internal consistency [41].

#### Stigma scale for receiving psychological help (SSRPH)

The SSRPH [18] is a five-item measure examining individual perceptions from society regarding receiving psychological help (i.e., public stigma). Items are rated from 0 (Strongly Disagree) to 3 (Strongly Agree), with higher scores indicating greater public stigma. For example, participants rated the item, “People tend to like less those who are receiving professional psychological help.” The Hungarian translation was derived from Kiss and colleagues (2020) and exhibited good internal consistency [42].

#### The acceptance and action questionnaire, version 2 (AAQ-II)

The AAQ [43] is a 7-item measure assessing EA. Items are rated on a 7-point Likert scale with higher scores indicating greater EA. For example, participants rated the statement, “I’m afraid of my feelings,” from 1 (Never True) to 7 (Always True). The Hungarian translation exhibits good psychometric properties [44].

#### Self-stigma of seeking help (SSOSH)

The SSOSH [45] is a 10-item measure assessing self-stigma associated with seeking psychological help. Items are rated from 1 (Strongly Disagree) to 5 (Strongly Agree), with higher scores indicating greater self-stigma. For example, participants rated the statement, “I would feel inadequate if I went to a therapist for psychological help.” The English version of the SSOSH exhibits good psychometric properties [45]. The SSOSH was translated into Hungarian by a bilingual professional in psychiatry. It was then back translated into English by another psychiatry professional. The back translated version was compared with the original English version by a third member of the research team, and discrepancies were discussed among the group of three translators. A sample of five native Hungarian speakers then read the Hungarian-translated SSOSH for concept checking. Appendix A provides the full Hungarian translation.

#### Beck’s Depression Inventory-shortened version (BDI-H)

The Beck Depression Inventory 2nd Edition (BDI-II) [46] is a 21-item self-report measure assessing somatic, affective, and behavioral symptoms (e.g., “I am too tired to do anything”) of depression over the prior 2 weeks. Items are rated on a 0-to-3-point scale and summed to yield a total score ranging from 0 (no symptoms) to 63 (very severe symptoms). The BDI-II was converted into a 9-item Hungarian version by Rózsa (2001) and exhibited good internal consistency and reliability [47].

#### State trait anxiety inventory (STAI)

The STAI is a 40-item self-report measure assessing current and trait-level anxiety using a Likert scale from 1 (not at all) to 4 (very much so) [48], with higher scores indicating greater anxiety symptoms. Given our interest in anxiety as a trait-level construct, we used the 20-item trait anxiety subscale. For example, participants rated the statement, “I worry too much over something that really does not matter.” We used the Hungarian version of the STAI trait subscale developed by Sipos (1983), which evidenced good reliability and validity [49].

### Data analysis

#### Central tendency and internal consistency of study measures

Central tendency and internal consistency analyses were conducted in R Studio. To examine internal consistency, we used both Cronbach’s alpha and McDonald’s omega. McDonald’s omega employs a factor analytic approach, whereas Cronbach’s alpha is primarily based on item correlations. McDonald’s omega has demonstrated greater robustness to deviations from the aforementioned assumptions, making it generally a more appropriate measure of internal consistency [50].

### Network analysis

Descriptive and network analyses were conducted in R Studio. As there were 3.5% missing values and missingness was at random per Little’s test of missing completely at random (MCAR; *Χ*^2^(92) = 101.00, *p* = .25), missing data was addressed using the default option on the bootnet package’s estimate Network function [51]. We used a network analytic approach to examine the associations between demographics, clinical characteristics, stigma, and exposure to mental health. Graphical LASSO models were conducted using the qgraph package [52–53] using the EBICglasso function [54–55] to examine the regularized partial correlation (edges) between variables (nodes). A penalty is applied to correlations close to zero, such that likely only more meaningful edges are retained.

Centrality parameters (e.g., strength, closeness, and betweenness) for the network were calculated using qgraph [52] to determine the relative importance of each node. Correlation stability coefficients were calculated using bootnet [56] to determine the stability of centrality parameters (e.g., strength, closeness, and betweenness). Coefficients greater than 0.50 indicate adequate stability, with lower stability suggesting that the network is sensitive to sampling changes. As the correlation stability coefficient was poor for closeness (0.05) and betweenness (0.05) and are considered less stable [57], we omitted these indices and focused our interpretation on strength centrality (i.e., the sum of the absolute value of all a node’s edges) which exhibited a good correlation stability coefficient (Strength = 0.75; see Figure S1 in Supplemental Materials). Differences in strength centrality (calculated at *p* = 0.05 level) were estimated using the differenceTest function in the R package, bootnet [56]. Finally, confidence intervals around edge weights were calculated to examine the accuracy of edges in the network. To examine the stability of strength centrality, centrality indices were recalculated after dropping increasing percentages of participants. A detailed explanation of network stability is available elsewhere [57].

## Results

**Figure 1.**
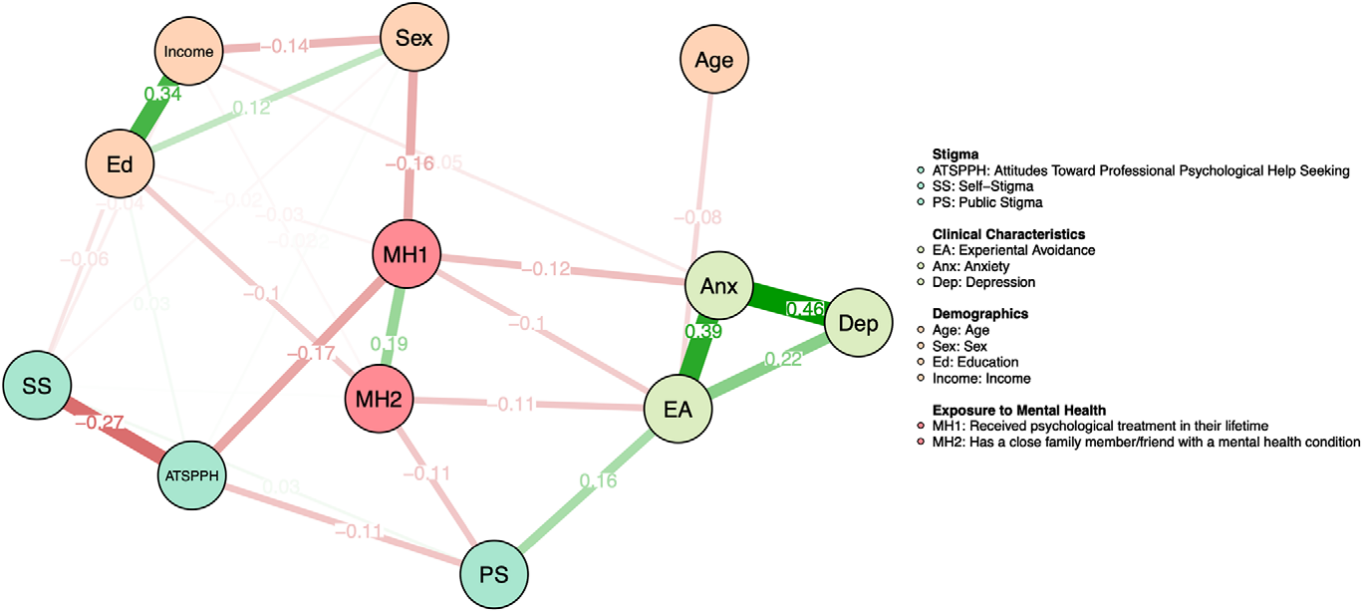
Network consisting of relationships between stigma, clinical characteristics, demographics, and exposure to mental health. Negative correlations are represented in red, and positive correlations are represented in green, with thicker lines representing stronger partial correlations.

**Figure 2.**
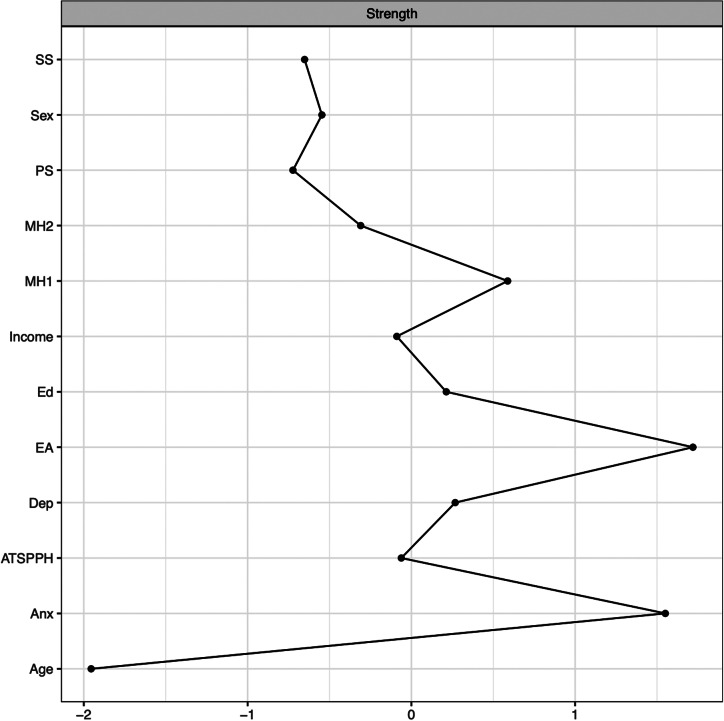
Strength Centrality Plot. *Note.* Higher scores are indicative of greater centrality in the network. SS = Self-stigma; PS = Public Stigma; MH2 = Has a close family member/friend with a mental health condition; MH1 = Received psychological treatment in their lifetime; Ed = Education; EA = Experiential Avoidance; Dep = Depression; ATSPPH = Attitudes Toward Seeking Professional Psychological Help; Anx = Anxiety.

Central tendency, dispersion, and internal consistency of study measures can be found in Table 2. The network is represented in Figure 1. As shown in Figure 2, EA and anxiety demonstrated the greatest strength centrality at 1.72 and 1.55, respectively, and exhibited significantly greater strength centrality than all other nodes in the network, though not from one another (see Supplemental Figure S2 for the difference in degree centrality among all nodes). After controlling for all other nodes in the network, the largest associations that remained were as follows: greater anxiety was associated greater depression (0.46), greater EA (.39), and having received psychological treatment in their lifetime (−0.12); greater EA was associated with having greater public stigma (0.16), having a family member or friend with a mental health condition (−0.11), having received psychological treatment in their lifetime (−0.10), and greater depression (0.22); more positive attitudes toward seeking help was associated with lower self-stigma (−0.27), lower public stigma (−0.11), and having received psychological treatment in their lifetime (−0.17); female sex was associated with lower income (−0.14), having received psychological treatment in their lifetime (−0.16), and higher education (0.12); having a close family member or friend with a mental condition was associated with having received psychological treatment in their lifetime (0.19) and greater public stigma (−0.11). The full list of edge weights is provided in Table 3. All nonregularized partial correlations are shown in Supplemental Table 1. Edge weight stability tests revealed small to moderate confidence intervals around edge weights (see Figure S3 in Supplemental Materials).

**Table 2.** Central tendency and dispersion of study measures

	Scale	M	SD	Cronbach’s Alpha	McDonald’s Omega
ATSPPH	Attitudes Toward Seeking Professional Psychological Help–Short Form	18.23	4.78	0.69	0.70
SS	Self–Stigma of Seeking Help	19.30	6.08	0.64	0.57
PS	Stigma Scale for Receiving Psychological Help	5.00	2.88	0.78	0.79
EA	The Acceptance and Action Questionnaire, Version 2	17.50	9.06	0.92	0.92
Anx	State Trait Anxiety Inventory–Trait Scale	42.40	10.00	0.87	0.89
Dep	Beck’s Depression Inventory–Shortened Version	12.75	4.01	0.83	0.84

Abbreviations: M = mean; SD = standard deviation; ATSPPH = attitudes toward seeking professional psychological help; SS = self-stigma; PS = public stigma; EA = experiential avoidance; Anx = anxiety; Dep = depression.

**Table 3.** Edge weights from partial correlation network

	ATSPPH	SS	PS	EA	Anx	Dep	Age	Sex	Education	Income	MH1	MH2
ATSPPH	0.00	−0.27	−0.11	0.00	0.00	0.00	0.00	0.02	0.03	0.00	−0.17	0.00
SS	−0.27	0.00	0.03	0.01	0.00	0.00	0.00	−0.02	−0.06	−0.04	0.00	0.00
PS	−0.11	0.03	0.00	0.16	0.00	0.00	0.00	0.00	0.00	0.00	0.00	−0.11
EA	0.00	0.01	0.16	0.00	0.39	0.21	−0.08	0.00	0.00	0.00	−0.10	−0.11
Anx	0.00	0.00	0.00	0.39	0.00	0.46	0.00	0.01	0.00	−0.05	−0.12	0.00
Dep	0.00	0.00	0.00	0.22	0.46	0.00	0.00	0.00	0.00	0.00	0.00	0.00
Age	0.00	0.00	0.00	−0.08	0.00	0.00	0.00	0.00	0.00	0.00	0.00	0.00
Sex	0.02	−0.02	0.00	0.00	0.01	0.00	0.00	0.00	0.12	−0.14	−0.16	0.00
Education	0.03	−0.06	0.00	0.00	0.00	0.00	0.00	0.12	0.00	0.34	−0.03	−0.10
Income	0.00	−0.04	0.00	0.00	−0.05	0.00	0.00	−0.14	0.34	0.00	0.00	−0.02
MH1	−0.17	0.00	0.00	−0.10	−0.12	0.00	0.00	−0.16	−0.03	0.00	0.00	0.19
MH2	0.00	0.00	−0.11	−0.11	0.00	0.00	0.00	0.00	−0.10	−0.02	0.19	0.00

Abbreviations: ATSPPH = attitudes toward seeking professional psychological help; SS = self-stigma; PS = public stigma; EA = experiential avoidance; Anx = anxiety; Dep = depression; MH1 = received psychological treatment in their lifetime; MH2 = has a close family member/friend with a mental health condition.

## Discussion

The present study used a network approach to examine the relationships between demographic variables, stigma, exposure to mental health, clinical symptoms, and EA in a sample of Hungarians visiting their primary care provider. Results indicated that EA and anxiety were the most central nodes in the network. Greater EA was associated with greater public stigma, anxiety, depression, and having a family member or friend with a mental health condition. More positive attitudes toward seeking help were associated with lower self-stigma, public stigma, and having received psychological treatment in their lifetime. Being female was associated with lower income, higher education, and having received psychological treatment in their lifetime. Finally, having a family member or friend with a mental health condition was associated with having received psychological treatment in their lifetime and greater public stigma. Findings highlight important targets (e.g., EA) that may play an important role in conceptualizations of stigma and help-seeking.

In accordance with research on the Internalized Stigma Model [38], EA was the most central node in the network, indicating its high connectedness with other nodes in the network and thereby, its relevance as a potential target to reducing stigma and increasing help-seeking. Previous research suggests that EA moderates the mediation of self-stigma on public stigma and intentions to seek help [38]. Extending this, our network suggests that EA may add explanatory value to previously researched associations between demographics and clinical variables with stigma. For example, prior research found associations between younger age and higher personal stigma [26], and greater clinical symptoms with higher perceived and self-stigma [33]. In the present network, age, anxiety, and depression were all indirectly associated with public stigma through EA. These findings suggest that EA may drive the association between clinical and demographic characteristics and stigma found in other studies. Future research should employ longitudinal designs to test EA as a mediator of the associations between clinical symptoms and public stigma and demographic variables and public stigma.

The association between EA and public stigma suggests that EA may be a relevant target in stigma reduction interventions. Interventions targeting EA, such as Acceptance and Commitment Therapy (ACT), may be beneficial. Indeed, research has found that a brief ACT workshop was effective at reducing mental health stigma at 1-month follow up [58]. ACT has also been used to reduce self-stigma among individuals with substance use disorder [59]. Despite the efficacy of these interventions, ACT-based treatment protocols are not commonplace in Hungary, with only one known study examining the efficacy of ACT among a small sample of Hungarian inmates [60].

Consistent with prior research, our findings suggest that stigma is an important factor in mental health help-seeking, even when accounting for covariates, evidenced through the retained associations between lower self-stigma and more positive attitudes toward seeking psychological help, lower public stigma and more positive attitudes toward seeking psychological help, and having received prior treatment and more positive attitudes toward seeking psychological help. These findings align with research suggesting that mental health stigma is a moderate barrier to help-seeking [5], and calls for research on interventions to increase help-seeking by targeting stigma and its correlates (e.g., EA). Moreover, while associations between public stigma and self-stigma typically emerge in cross-sectional stigma models (e.g., [20, 27, 38], and longitudinal research finds that public stigma leads to the internalization of self-stigma [21], only a very small association emerged between public stigma and self-stigma in the present study. This suggests that factors beyond societal attitudes play a significant role in shaping individual’s perceptions of themselves and their mental health. It may be due to the characteristics of the sample (patients of primary care providers), cultural differences in the Hungarian sample (i.e., public stigma may not be internalized as self-stigma in this population), and a more robust inclusion of covariates in the present study. To address this, future research should examine differences in the internalization of public stigma as self-stigma across diverse populations. Moreover, the greater strength centrality of self-stigma and its lack of association with public stigma suggests that it may be a more relevant target in stigma-reducing interventions. As such, future research may benefit from examining ways to target self-stigma directly. For example, interventions targeting self-stigma, such as “Coming Out Proud” [61], may be of particular benefit for reducing self-stigma.

Greater public stigma was associated with having a close family member or friend with a mental health condition. This is consistent with research suggesting that family members may have increased public stigma as a result of experiencing the burden of having a family member with a mental health condition [34]. As noted by Corrigan & Nieweglowski (2019), research is needed to identify how burden contributes to public stigma within specific friend and familial roles (e.g., friends versus parents) and across cultures. As the present study did not operationalize having a close family member with a mental health condition as constituting only immediate family members, it is likely the association with public stigma encompasses relationships beyond immediate family members. Furthermore, having a family member or friend with a mental health condition was associated with greater EA. It is possible that having witnessed the negative effects of mental illness on one’s life, friends and family may be more inclined to avoid negative emotions and experiences that are typically associated with having a mental health condition. Additionally, friends and family members may experience greater shame, stress, and burnout, leading to greater efforts to avoid experiencing these negative feelings.

When controlling for other variables in the network, women were more likely to have higher education but lower income. This is consistent with prior research showing a gender pay gap in Hungary, with the average graduated woman being paid 16% less than the average graduated man [62]. Additionally, the network analysis revealed that women were more likely to receive treatment for their mental health than men, irrespective of clinical symptoms. This is consistent with research from several countries around the world, finding that women have more positive attitudes toward seeking psychological help [23, 63, 64] and are more likely to seek psychological help than men (e.g., [65]). Taken together, these findings further support an established discrepancy and need for gender and income equality for women in Hungary. Findings also highlight the need for targeted approaches to increase help-seeking in men. Interventions such as the Man Up documentary, a film discussing the association between beliefs about masculinity, men’s mental health, and suicidal thoughts and behaviors, have been efficacious at increasing help-seeking behavior in men [66] and may warrant more attention among the Hungarian community.

The present study has a number of limitations. First, as a cross-sectional study, we cannot make causal inferences. Future research using longitudinal data to examine the directionality of the associations identified in the present study would be beneficial. Second, while all patients attending their primary care offices on the given study day were invited to participate in the study, it is likely that those with more positive attitudes toward mental health had a greater inclination to participate, potentially skewing stigma ratings. Social desirability bias may have also led to more favorable stigma ratings. Future research would benefit from a larger, randomly selected sample. Third, internal consistency measures were acceptable for all scales except SSOSH, which fell slightly below the acceptable range. For the Hungarian version of the SSOSH, a more thorough investigation of its psychometric properties using factor analysis is recommended for future research. Fourth, as mental health literacy varies considerably across Hungary and the practices selected do not encompass the entirety of this variability, we are limited in our ability to make generalizations about the Hungarian population. Moreover, though our case-dropping bootstrap and prior research on optimal sample sizes in networks with 20 nodes or less [67] suggest that our sample size was adequate, increasing the sample size would improve network estimates and detection of differences in centrality [57]. Lastly, as the sample consisted of those visiting their primary care provider, it is possible that rates of treatment utilization were higher than in the broader community, as those visiting their primary care providers may overrepresent those experiencing chronic mental or physical illnesses.

In summary, findings highlight a need for research on stigma and help-seeking outside of American and Western European countries. Findings suggest that while some aspects of existing stigma models may be consistent in countries like Hungary, other aspects may diverge. Future studies would benefit from path analysis of longitudinal data in order to examine the directionality of associations identified in the present study.

## Supporting information

Swisher et al. supplementary material Swisher et al. supplementary material

## Data Availability

The data that support the findings of this study are available on request from the corresponding author, Dorottya Őri.

## Supplementary material

The supplementary material for this article can be found at http://doi.org/10.1192/j.eurpsy.2024.1772.

## Appendix A

### Self-Stigma of Seeking Help (SSOSH; Vogel et al., 2006) – Hungarian Translation

Ebben a részben néhány állítást olvashat. Kérjük, jelölje meg azt, hogy mennyire ért egyet az adott állítással. Használja a következő skálát.

**Table:**

	Egyáltalán nem értek egyet		Egyet is értek és nem is értek egyet		Nagyon egyetértek
1. Alkalmatlannak érezném magam, ha terapeutához fordulnék pszichológiai segítségért.	1	2	3	4	5
2. Az önbizalmamat NEM fenyegetné, ha szakszerű segítséget kérnék.	1	2	3	4	5
3. Ha pszichológiai segítséget kérnék, kevésbé érezném magam intelligensnek.	1	2	3	4	5
4. Az önbecsülésem nőne, ha terapeutával beszélnék.	1	2	3	4	5
5. A magamról alkotott képem nem változna csak azért, mert úgy döntöttem, hogy terapeutát keresek fel.	1	2	3	4	5
6. Alacsonyabbrendűnek érezném magam, ha segítséget kérnék terapeutától.	1	2	3	4	5
7. Elfogadnám magamat, ha úgy dönthetnék, hogy szakembert keresek fel.	1	2	3	4	5
8. Ha elmennék terapeutához, kevésbé lennék elégedett magammal.	1	2	3	4	5
9. Az önbizalmam változatlan maradna, ha olyan problémában kérnék segítséget, amit magam nem tudok megoldani.	1	2	3	4	5
10. Rosszabb érzéseim lennének magammal kapcsolatban, ha nem tudnám megoldani a saját problémáimat	1	2	3	4	5
