# Supplementary material for: Understanding mental health help-seeking and stigma among Hungarian adults: A network perspective
Source: Eur Psychiatry. 2024 Sep 19;67(1):e52. doi: 10.1192/j.eurpsy.2024.1772 (PMC11457119; doi:10.1192/j.eurpsy.2024.1772)
Supplement: Swisher et al. supplementary material [file S0924933824017723sup001.zip › Fig S1.docx]

**
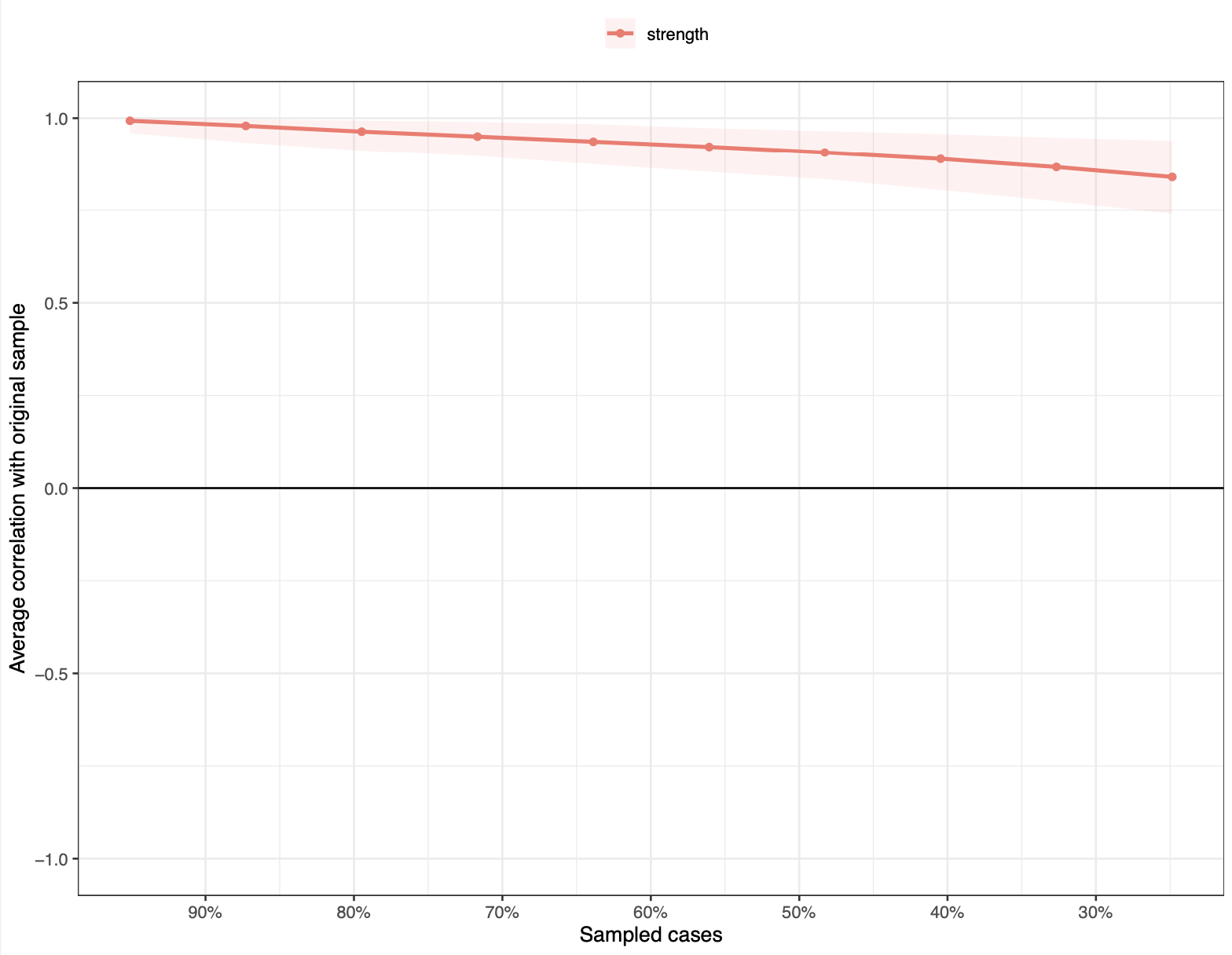
Figure S1.** Strength Centrality Stability

*Note.* Stability coefficients for strength centrality when using subsetting bootstrap.
