# Supplementary material for: Understanding mental health help-seeking and stigma among Hungarian adults: A network perspective
Source: Eur Psychiatry. 2024 Sep 19;67(1):e52. doi: 10.1192/j.eurpsy.2024.1772 (PMC11457119; doi:10.1192/j.eurpsy.2024.1772)
Supplement: Swisher et al. supplementary material [file S0924933824017723sup001.zip › Figure S2.docx]

**
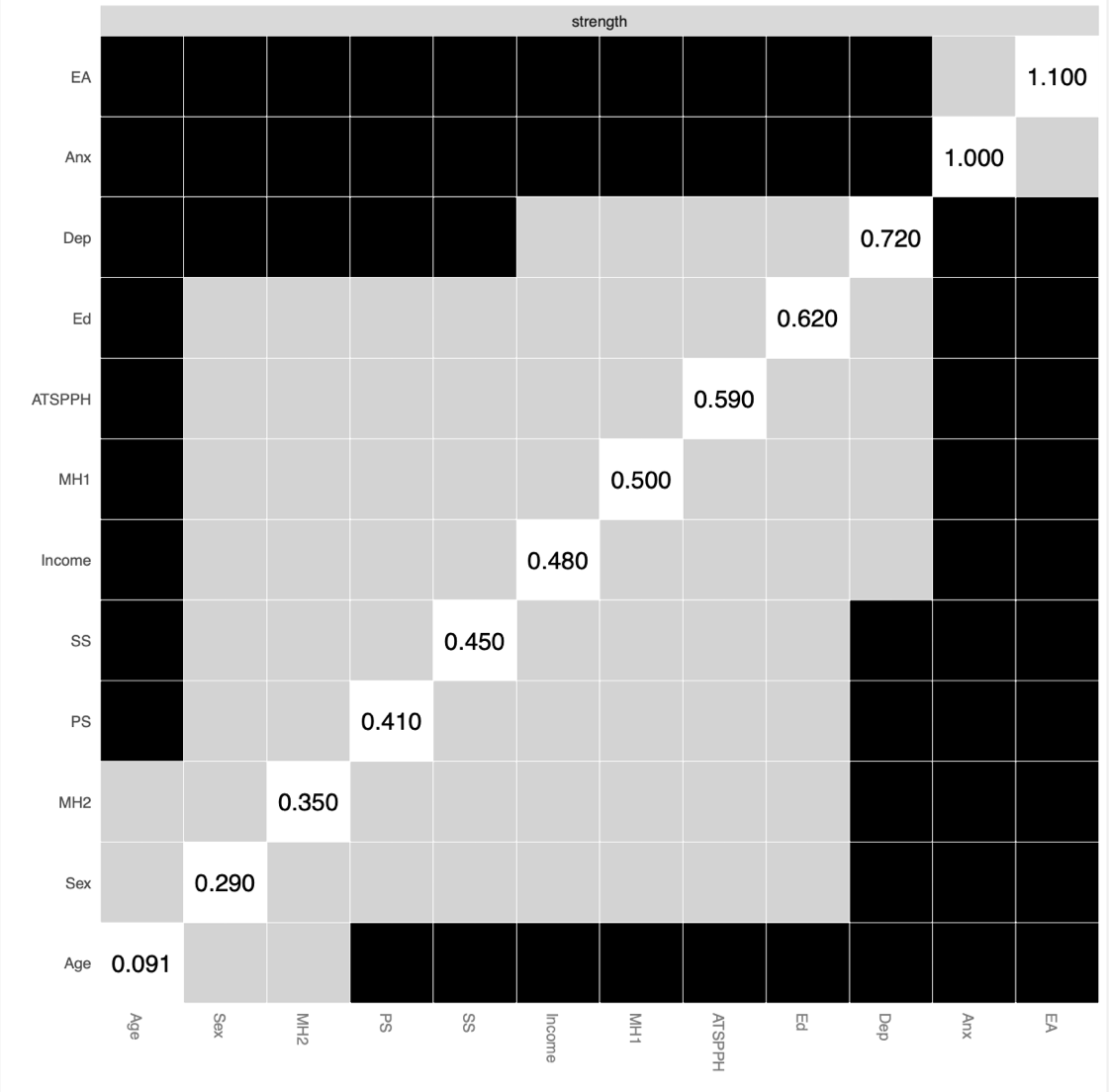
Figure S2.** Degree centrality difference test for the 12-item network.

*Note*. Gray boxes indicate that the strength centrality among nodes that do not significantly differ from one another. Black boxes indicate that the strength centrality among nodes that do significantly differ from one another. SS = Self-stigma; PS = Public Stigma; MH2 = Has a close family member/friend with a mental health condition; MH1 = Received psychological treatment in their lifetime; Ed = Education; EA = Experiential Avoidance; Dep = Depression; ATSPPH = Attitudes Toward Seeking Professional Psychological Help; Anx = Anxiety
