# Supplementary material for: Understanding mental health help-seeking and stigma among Hungarian adults: A network perspective
Source: Eur Psychiatry. 2024 Sep 19;67(1):e52. doi: 10.1192/j.eurpsy.2024.1772 (PMC11457119; doi:10.1192/j.eurpsy.2024.1772)
Supplement: Swisher et al. supplementary material [file S0924933824017723sup001.zip › Figure S3.docx]

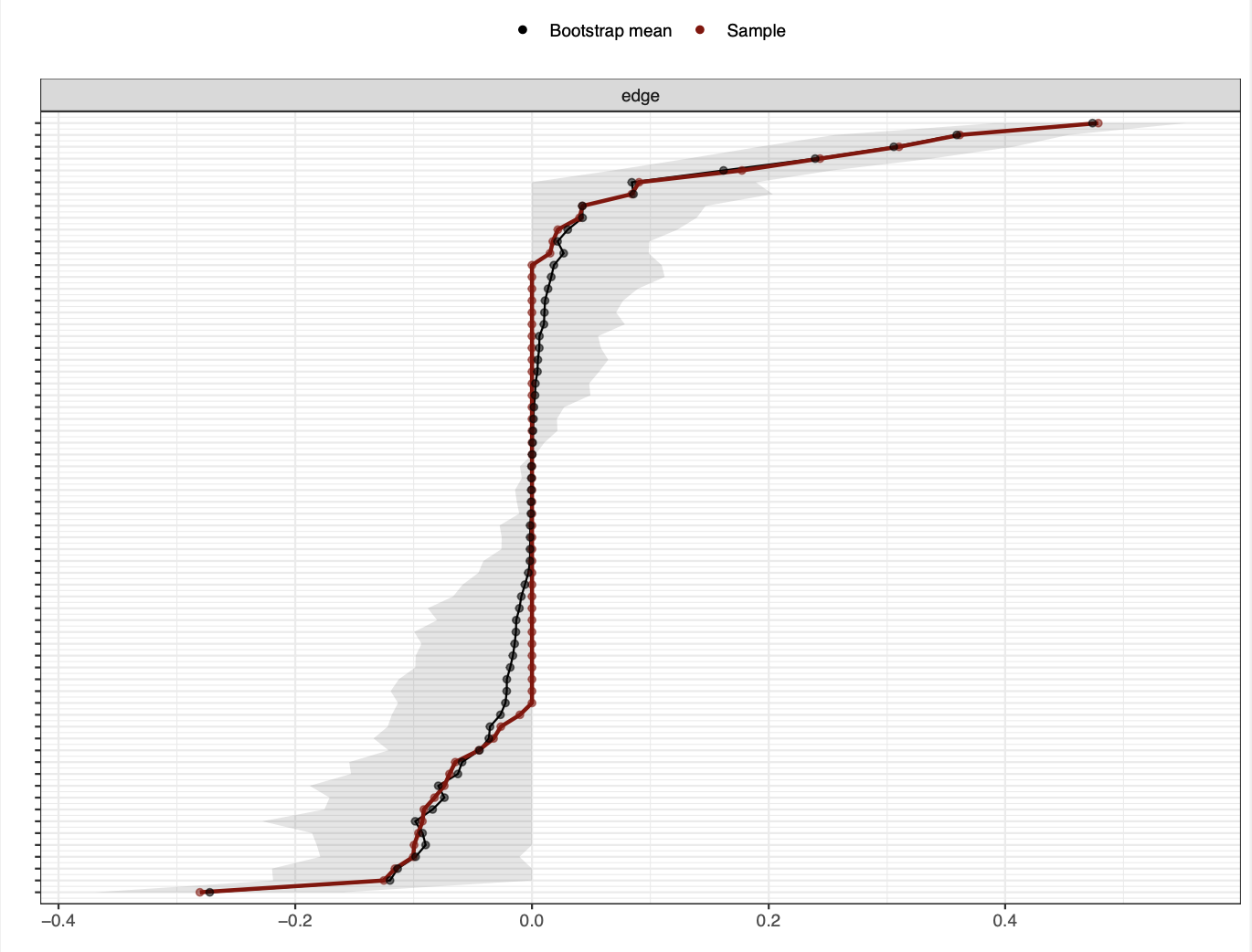
**Figure S3.** Bootstrapped confidence intervals of the network edge weights

*Note*. The red line indicates the edge weight values and the gray area the 95% CIs.
