# Supplementary material for: Understanding mental health help-seeking and stigma among Hungarian adults: A network perspective
Source: Eur Psychiatry. 2024 Sep 19;67(1):e52. doi: 10.1192/j.eurpsy.2024.1772 (PMC11457119; doi:10.1192/j.eurpsy.2024.1772)
Supplement: Swisher et al. supplementary material [file S0924933824017723sup001.zip › Rev_Supplemental Table 1 (NONRegularized Edge Weights).docx]

Supplemental Table 1

*Nonregularized Partial Correlations between Nodes*

|  | ATSPPH | SS | PS | EA | Anx | Dep | Age | Sex | Education | Income | MH1 | MH2 |
| --- | --- | --- | --- | --- | --- | --- | --- | --- | --- | --- | --- | --- |
| ATSPPH | 1.00 | -0.39 | -0.23 | 0.00 | 0.01 | -0.07 | -0.06 | 0.17 | 0.18 | 0.17 | 0.18 | 0.13 |
| SS | -0.39 | 1.00 | 0.18 | 0.13 | 0.11 | 0.10 | 0.20 | -0.15 | -0.20 | -0.15 | -0.20 | -0.17 |
| PS | -0.23 | 0.18 | 1.00 | 0.34 | 0.23 | 0.21 | -0.01 | -0.07 | 0.02 | -0.07 | 0.02 | -0.11 |
| EA | 0.00 | 0.13 | 0.34 | 1.00 | 0.72 | 0.64 | -0.22 | 0.08 | -0.01 | 0.08 | -0.01 | -0.08 |
| Anx | 0.01 | 0.11 | 0.23 | 0.72 | 1.00 | 0.75 | -0.16 | 0.18 | -0.01 | 0.18 | -0.01 | -0.18 |
| Dep | -0.07 | 0.10 | 0.21 | 0.64 | 0.75 | 1.00 | -0.12 | 0.04 | -0.06 | 0.04 | -0.06 | -0.10 |
| Age | -0.06 | 0.02 | -0.01 | -0.22 | -0.16 | -0.12 | 1.00 | 0.10 | 0.07 | 0.10 | 0.07 | -0.08 |
| Sex | 0.18 | -0.15 | -0.07 | 0.08 | 0.18 | 0.04 | 0.10 | 1.00 | 0.20 | 1.00 | 0.20 | -0.22 |
| Education | 0.18 | -0.20 | 0.02 | -0.01 | -0.01 | -0.06 | 0.07 | 0.20 | 1.00 | 0.20 | 1.00 | 0.44 |
| Income | 0.13 | -0.17 | -0.11 | -0.08 | -0.18 | -0.10 | -0.08 | -0.22 | 0.44 | -0.22 | 0.44 | 1.00 |
| MH1 | -0.30 | 0.14 | -0.11 | -0.38 | -0.39 | -0.31 | 0.03 | 0.30 | -0.19 | -0.30 | -0.19 | 0.01 |
| MH2 | -0.12 | 0.06 | -0.26 | -0.33 | -0.19 | -0.16 | 0.04 | -0.16 | -0.24 | -0.16 | -0.24 | -0.16 |

*Note.* ATSPPH = Attitudes Toward Seeking Professional Psychological Help; SS = Self-stigma; PS = Public Stigma; EA = Experiential Avoidance; Anx = Anxiety; Dep = Depression; MH1 = Received psychological treatment in their lifetime; MH2 = Has a close family member/friend with a mental health condition
